# The biosynthetic pathway to ossamycin, a macrocyclic polyketide bearing a spiroacetal moiety

**DOI:** 10.1371/journal.pone.0215958

**Published:** 2019-04-30

**Authors:** Oksana Bilyk, Markiyan Samborskyy, Peter F. Leadlay

**Affiliations:** Department of Biochemistry, University of Cambridge, Cambridge, United Kingdom; Universite Paris-Sud, FRANCE

## Abstract

Ossamycin from *Streptomyces hygroscopicus* var. *ossamyceticus* is an antifungal and cytotoxic polyketide and a potent inhibitor of the mitochondrial ATPase. Analysis of a near-complete genome sequence of the ossamycin producer has allowed the identification of the 127-kbp ossamycin biosynthetic gene cluster. The presence in the cluster of a specific crotonyl-CoA carboxylase/reductase homologue suggests that the 5-methylhexanoate extension unit used in construction of the macrocyclic core is incorporated intact from the unusual precursor isobutyrylmalonyl-CoA. Surprisingly, the modular polyketide synthase uses only 14 extension modules to accomplish 15 cycles of polyketide chain extension, a rare example of programmed iteration on a modular polyketide synthase. Specific deletion of genes encoding cytochrome P450 enzymes has given insight into the late-stage tailoring of the ossamycin macrocycle required for the attachment of the unusual 2,3,4,6-deoxyaminohexose sugar l-ossamine to C-8 of the ossamycin macrocycle. The ossamycin cluster also encodes a putative spirocyclase enzyme, OssO, which may play a role in establishing the characteristic spiroketal moiety of the natural product.

## Introduction

**Fig 1:**
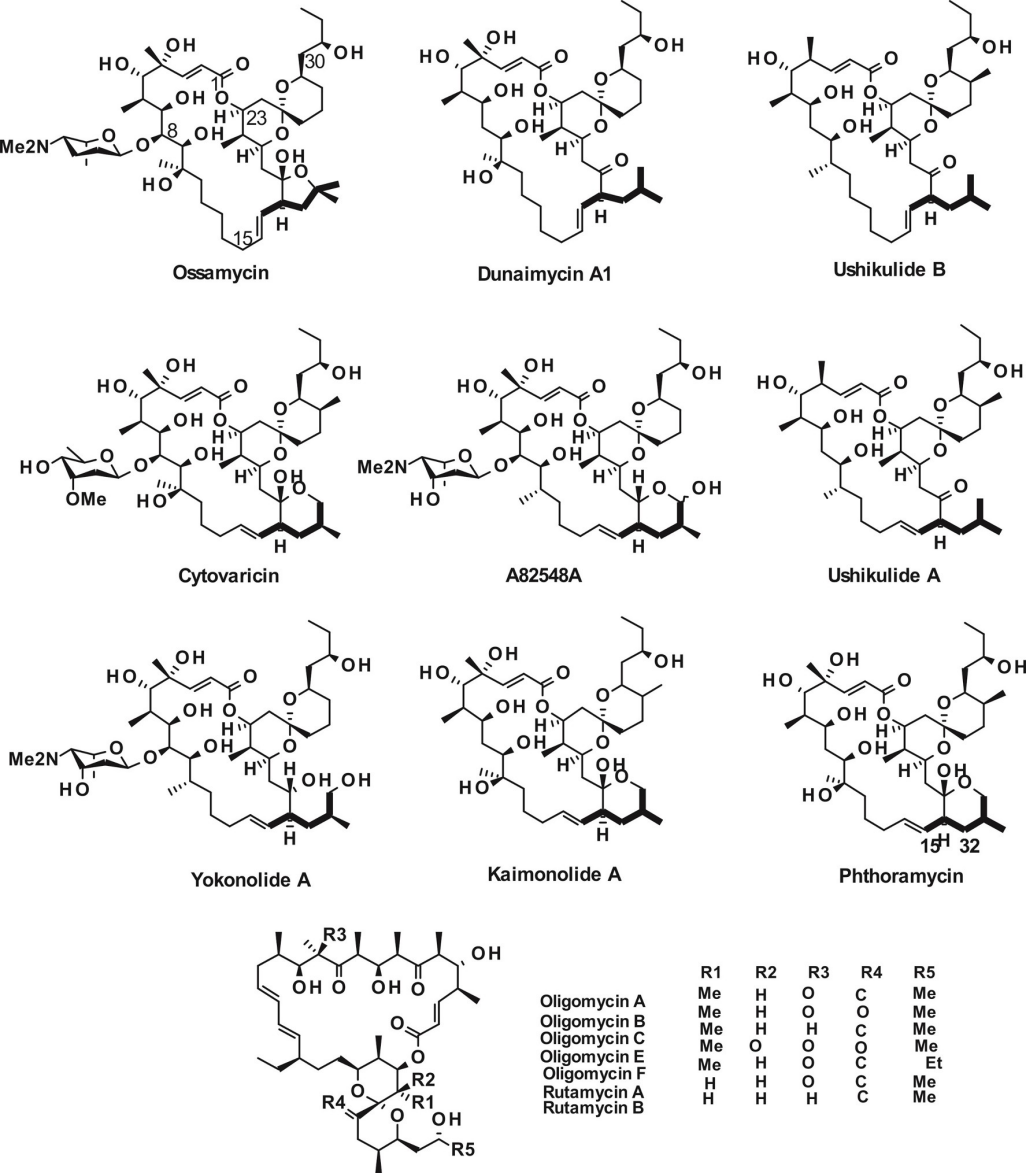
Chemical structures of the 22- to 26-membered macrocyclic polyketides discussed in this work.

Ossamycin from *Streptomyces hygroscopicus* var. *ossamyceticus* is a cytotoxic polyketide first reported in 1965 [1]. It is one of a family of 22- to 26-membered macrocyclic polyketides whose structural hallmark is a 6,6-spiroacetal (1,7-dioxaspiro[5,5]-undecanyl) moiety welded to one side of the macrocycle (Fig 1). The best-known and most widely-studied of these compounds are the 26-membered oligomycins/rutamycins [2], potent inhibitors of the mitochondrial F_1_F_0_-ATPase [3]. Various other macrocyclic polyketides related to the oligomycins/rutamycins have been isolated (Fig 1) by screening for antifungal, immunosuppressive or cytotoxic bioactivity, including for example the 24-membered dunaimycins [4], and the 22-membered cytovaricin [5], A82548A (also known as yokonolide B) [6], yokonolide A [7], phthoramycin [8], ushikulides A and B [9] and kaimonolide A [10], all produced by *Streptomyces* spp. and many of them by different strains of *S*. *diastatochromogenes*. The chemical structures of these compounds have been established by detailed NMR analysis. Also, assignment of relative and absolute stereochemistry at the multiple stereocentres has been achieved for representative compounds through single-crystal X-ray crystallography and elegant total synthesis [6, 11–14]. This has disclosed the striking structural consistency between the compounds of Fig 1. Even more remarkably, comparison [11,13] of the X-ray crystal structures of cytovaricin and rutamycin A has shown that, despite the differences in ring size and in substituents, their aglycones are superimposable. Consistent with this, cytovaricin and ossamycin act as selective cytotoxic agents through inhibition of the F_o_ component of F_1_F_0_-ATPases, the known target of oligomycin [15].

The structural relationships established between the members of this family of complex polyketides suggest a common pathway for their biosynthesis. As part of the whole-genome sequencing of the avermectin producer *Streptomyces avermitilis*, the 95-kbp biosynthetic gene cluster for oligomycin A has been sequenced [16], and shown to encode a canonical modular type I polyketide synthase (PKS) consisting of seven multifunctional subunits, together with regulatory genes and genes encoding enzymes for production of the C-4 building block ethylmalonyl-CoA. In contrast, the biosynthesis of the 22- and 24-membered spiroacetal family of macrocyclic polyketides has remained obscure. We have now used a genome-sequencing approach to identify and analyse the biosynthetic gene cluster for ossamycin A, and we show that the ossamycin modular PKS is one of the rare examples so far uncovered in which programmed iteration is required to assemble its product. This provides new insight into the biosynthetic relationship between 22- and 24-membered spiroacetal compounds. We have also clarified the origin of the unusual 5-methylhexanoate building block for the PKS, and have used selective mutation to probe the role of individual cytochrome P450 enzymes in post-PKS oxidation of the ossamycin macrocycle. The product of the gene *ossO* has significant sequence similarity to the uncharacterised enzyme OlmO in the oligomycin biosynthetic gene cluster, and may represent a novel spirocyclase enzyme involved in construction of the 6,6-spiroacetal.

## Results and discussion

### Identification and analysis of a candidate gene cluster for ossamycin biosynthesis in *Streptomyces hygroscopicus* var. *ossamyceticus* NRRL B-3822

**Fig 2:**
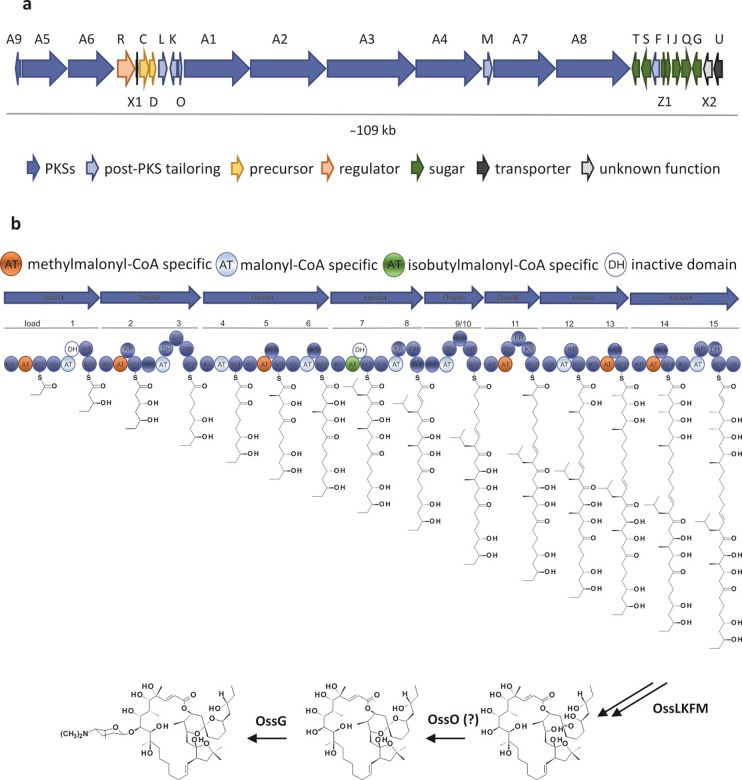
Ossamycin biosynthetic gene cluster in *S*. *hygroscopicus* var. *ossamyceticus* DSM40824: (a) arrangement of the *oss* cluster; (b) proposed biosynthetic pathway of ossamycin.

The ossamycin producer was sequenced using the Illumina platform and the final genome assembly contained essentially all the sequence of the *S*. *ossamyceticus* linear chromosome within a single high quality scaffold of 10,033,004 bp. This is approximately 0.7 Mbp longer than the genome size previously estimated from shotgun sequencing of this strain (1008 scaffolds) (accession number NZ_LIQX00000000). Biosynthetic gene clusters were initially detected using the program AntiSMASH [17], which revealed 29 clusters, only one of which (*oss*) encoded a modular PKS of appropriate size to be considered a candidate for ossamycin biosynthesis. Manual inspection and curation using the program Artemis [18] led to the definition of the cluster as housing 27 contiguous open reading frames (ORFs) within a DNA region of 127 kbp (accession number MH7636247). The deduced organisation of the genes in the cluster is shown in Fig 2A, and the predicted function of each gene is given in Table 1. The limits of the cluster are provisionally taken to be where the flanking genes are clearly involved in primary metabolism. The *oss* cluster contains eight giant modular PKS genes (*ossA1-ossA8*), which together govern assembly of the polyketide backbone from carboxylic acid precursors [19, 20]. Other genes in the cluster clearly encode enzymes with significant sequence similarity to authentic enzymes of deoxysugar biosynthesis and attachment, as expected from the structure of ossamycin; and genes likely to be involved in the post-assembly oxidations that occur around the macrocycle. In fact, almost all the genes in the cluster can be ascribed plausible roles in the biosynthesis, as described below.

**Table 1:** Predicted functions of the proteins encoded in the ossamycin gene cluster.

Length (aa)	Name	Proposed function	NCBI hit (identity)	Acc. Number
260	OssA9	Thioesterase	*S*. sp. MA5143a (94%)	WP_107466330.1
2192	OssA5	Polyketide synthase	*S*.*s* sp. MA5143a (87%)	WP_107466329.1
2214	OssA6	Polyketide synthase	EbeB, *K*. *aburaviensis* (56%)	AGY62754.1
865	OssR	LuxR family transcriptional regulator	*S*. *torulosus* (77%)	WP_055712771.1
69	OssX1	Hypothetical protein	*S*.*s* sp. MA5143a (85%)	WP_107466326.1
455	OssC	Crotonyl-CoA reductase	*S*. *torulosus* (98%)	WP_055712773.1
330	OssD	Ketoacyl-ACP synthase III	*S*. *bottropensis* (96%)	WP_005483819.1
404	OssL	Cytochrome P450	*S*. *bottropensis* (83%)	WP_020115058.1
404	OssK	Cytochrome P450	*S*. *bottropensis* (92%)	WP_005483816.1
176	OssO	Spirocyclase	OlmO, *S*. *avermitilis* (43%)	WP_010984323.1
3194	OssA1	Polyketide synthase	*S*. sp. cf124 (71%)	WP_107439495.1
3691	OssA2	Polyketide synthase	NimA1, *S*. *nanchangensis* (57%)	AAS46341.1
4306	OssA3	Polyketide synthase	TamAl *S*. sp. 307–9 (57%)	ADC79637.1
3227	OssA4	Polyketide synthase	TgaA *Sorangium cellulosum* (49%)	ADH04639.1
400	OssM	Cytochrome P450	*S*. sp. MA5143a (90%)	WP_107468069.1
3015	OssA7	Polyketide synthase	NimA1, *S*. *nanchangensis* (55%)	AAS46341.1
3594	OssA8	Polyketide synthase	*S*.*s* sp. MA5143a (84%)	WP_107467267.1
333	OssT	NDP-hexose-3-ketoreductase	ChlC4, *S*. *antibioticus* (58%)	AAZ77681.1
452	OssS	NDP-hexose-2,3-dehydratase	PgaS, *S*. sp. PGA64 (53%)	AHW57789.1
396	OssF	Cytochrome P450	*S*. sp. MA5143a (87%)	WP_107467265.1
198	OssZ1	dTDP-4-keto-6-deoxyhexose 3,5-epimerase	AknL, *S*. *galilaeus* (58%)	AAF70101.1
251	OssI	SAM-dependent methyltransferase	BusS, *Saccharopolyspora pogona* (61%)	AAY88936.1
380	OssJ	dTDP-4-amino-4,6-dideoxygalactose transaminase	*S*. sp. cf124 (83%)	SFN43901.1
439	OssQ	NDP-hexose 3,4-dehydratase	UrdQ, *S*. *fradiae* (81%)	AAF72550.1
413	OssGT	Glycosyltransferase	NivK, *S*. sp. Ls2151 (47%)	AGZ78380.1
421	OssX2	serine hydroxymethyltransferase	*S*. *torulosus* (98%)	WP_055716418.1
423	OssT	ABC transporter	*S*. *torulosus* (99%)	WP_055717176.1

### Analysis of the origin of the unusual 4-methylpentanoate extender unit

It is a conserved feature of the 22- and 24-membered macrocyclic spiroacetals (Fig 1) that 4-methylpentanoate is apparently incorporated as an unusual extender unit. In the ushikulides A and B and in dunaimycin A1 the sidechain remains intact, but in ossamycin and other compounds the sidechain is oxidised and may form a 5- or 6-membered hemiacetal ring in the final product of the pathway. The feeding of [1-^13^C]-isobutyrate to the phthoramycin-producing strain [8] confirmed that isobutyrate is the origin of the C4 unit in the sidechain at C-32, C-33, C-34 and C-35. Strikingly, a high enrichment of ^13^C was also found at C-15 when [1-^13^C]-isocaproate was fed, which provided the first evidence for use of isobutyrylmalonyl-thioester as an unusual extender unit [8]. Since then, use of this rare branched extension unit has also been detected in the divergolides [21,22] and ansalactams [23].

Two main pathways have been described for supply of such extended chain building blocks to modular PKS systems: well-characterised crotonyl-CoA reductase/carboxylase (CCR) enzymes which catalyse the reductive carboxylation of α,β-unsaturated acyl-CoA substrates to substituted malonyl-CoA derivatives of various chain lengths [24]; and a new pathway in which a biotin-dependent acyl-CoA carboxylase (ACC) directly converts medium-chain fatty acyl-CoA esters into the corresponding alkylmalonyl-CoA [25]. Inspection of the ossamycin cluster showed the presence of adjacent genes for the CCR homologue OssC and a FabH homologue OssD (Fig 2 and Table 1) which makes this route the obviously preferred one to consider [24, 26, 27]. FabH enzymes are related to 3-oxoacyl-ACP synthases in fatty acid synthesis and could catalyse the condensation of isobutyryl-CoA (derived in turn from branched-chain amino acid catabolism) with malonyl-CoA to form 4-methyl-3-oxo-pentanoate, which could be reduced and dehydrated by enzymes from fatty acid metabolism to provide 4-methyl-2-pentenoyl-CoA (4,4-dimethylcrotonyl-CoA) as a substrate for OssC. In support of this idea, the FabH-like DivS (60% sequence identity with OssD) and CCR DivR (69% identical with OssC) from the divergolide gene cluster, when heterologously co-expressed with the germicidin PKS, has been shown to confer production of specific germicidins containing the 4-methylpentanoate unit [21].

### The pathway to the rare deoxyaminosugar l-ossamine is encoded by six genes in the ossamycin cluster

**Fig 3:**
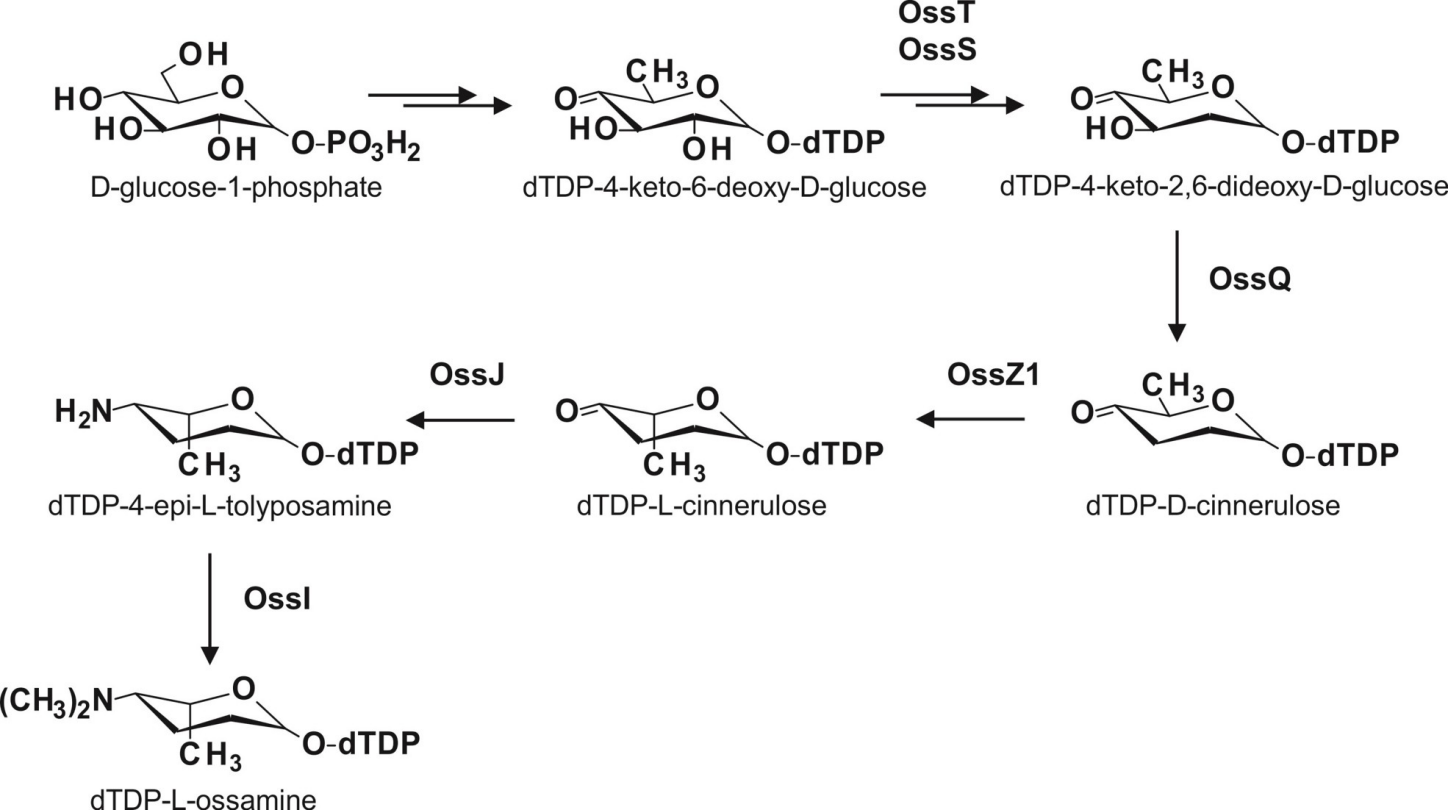
Biosynthesis of the L-ossamine.

Ossamycin differs from the other polyketide ATPase inhibitors in Fig 1 in that it bears the rare, highly reduced aminosugar l-ossamine attached to the ossamycin macrocycle at the C-8 position of the macrocycle. The ossamine moiety of ossamycin was originally reported to have the d- configuration [28] but X-ray crystallography of ossamycin later showed it to be in the l- configuration [6]. Subsequently, l-ossamine has been determined also to be a constituent of the aromatic polyketide frigocyclinone [29] and of spinosyn G, a minor component of the spinosyn complex from *Saccharopolyspora spinosa* [30,31]. The genes and enzymes for biosynthesis and attachment of specific deoxy(amino)hexose sugars to polyketide antibiotics have been intensively studied [32,33], and this allows roles in l-ossamine biosynthesis to be allocated to several genes in the *oss* cluster (Fig 3). The key early intermediate dTDP-4-keto-6-deoxy-d-glucose is formed from d-glucose-1-phosphate through the tandem action of dTDP-d-glucose synthase and dTDP-d-glucose 4,6-dehydratase. Genes encoding these two enzymes are missing from the ossamycin gene cluster and apparently are recruited from somewhere else in the genome. The conversion of dTDP-4-keto-6-deoxy-d-glucose into l-ossamine is proposed to involve, first, 2-deoxygenation catalysed by 2,3-dehydratase OssS and 3-ketoreductase OssT; followed by 3-deoxygenation catalysed by 3,4-dehydratase OssQ (assisted by an undefined reductase encoded elsewhere [33]) to provide dTDP-d-cinerulose (Fig 3) [34, 35]. Ensuing 5-epimerisation or 3,5-epimerisation is catalysed by OssZ1 (homologous to UrdZ1 in urdamycin biosynthesis) [34], followed by 4-transamination catalysed by OssJ, and then S-adenosylmethionine-dependent N-dimethylation catalysed by OssI to yield the glycosyl donor dTDP-l-ossamine. This is used as substrate by glycosyltransferase OssG to transfer l-ossamine to a specific hydroxy group previously installed at C-8 of the ossamycin aglycone. In the absence of biochemical evidence, it cannot be excluded that 5-epimerisation or 3,5-epimerisation actually occurs earlier in the pathway. If it occurred after 2-deoxygenation but before 3-deoxygenation, then dTDP-l-kedarosamine would be formed as an intermediate, and it is perhaps relevant that l-kedarosamine is the sugar found attached to C-8 in yokonolide A and in A82548A (yokonolide B) (Fig 1).

### Cytochrome P450 enzymes create late-stage diversity among 22- and 24-membered macrocyclic spiroacetals

The ossamycin polyketide backbone undergoes four stereospecific hydroxylations at C-4, C-8, and C-10 in the macrocycle; and at C-35 in the unusual sidechain, respectively. The only oxidative enzymes encoded in the *oss* cluster are the four cytochrome P450 enzymes OssL, OssK, OssM and OssF, with rather low (25–38%) mutual sequence identity (Fig 2 and Table 1), so it is tempting to suggest that each P450 catalyses a regiospecific hydroxylation. The natural spiroacetal compounds in Fig 1 differ markedly in the extent of their hydroxylation: the ushikulides show none at all; yokonolides A and B lack the hydroxy group at C-10; while phthoramycin and kaimonolide A lack the C-8 hydroxy group, without which glycosyl transfer cannot occur. The same is true of dunaimycin A1, though other dunaimycin congeners co-produced with it in *Streptomyces diastatochromogenes* are both hydroxylated and glycosylated at C-8 [4].

**Fig 4:**
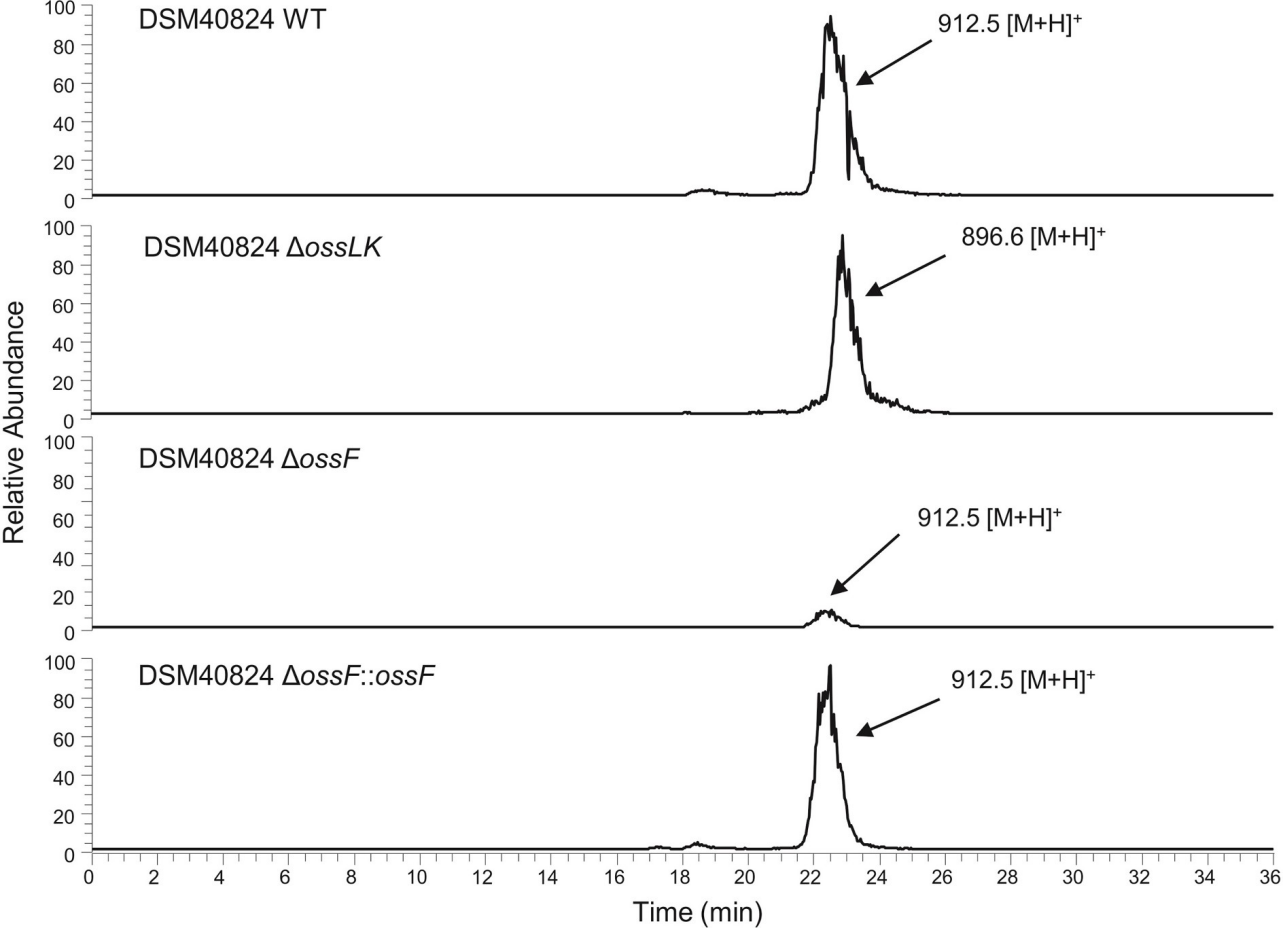
ESI-MS traces of the crude extracts from *S*. *hygroscopicus* var. *ossamyceticus* DSM 40824 (DSM40824 WT), mutant with the double deletion *ossLK* (DSM40824 *ΔossLK*), mutant with the deletion of *ossF* (DSM40824 *ΔossF*) and complementation (DSM40824 *ΔossF*::*ossF*).

To assess the consequences of perturbing hydroxylation steps in ossamycin biosynthesis, in-frame deletion of *ossF* and double-deletion of *ossL* and *ossK* were carried out as described in the Experimental section. It was expected that if any of these three enzymes were uniquely responsible for hydroxylation at C-8, then the attachment of ossamine at this position would also be prevented. LC-MS analysis of fermentation extracts of *S*. *ossamyceticus* Δ*ossF* showed that ossamycin production was not abolished, but it was reduced to less than 10% of the levels in the parent strain. Complementation of the mutant with a wild-type copy of the *ossF* gene fully restored ossamycin production (Fig 4). The simplest explanation of these results is that there is limited crosstalk between OssF and one of the other P450 enzymes acting in this pathway, so that one of OssK, L or M can partly replace OssF. Also, the adventitious activity of a cytochrome P450 not encoded within the *oss* cluster cannot be ruled out. LC-MS analysis of fermentation extracts of *S*. *ossamyceticus ΔossLK* (Fig 4) revealed complete loss of ossamycin production and the presence instead of a compound with the same retention time as ossamycin but with lower m/z = 896.5 [M+H]^+^. The observed mass corresponds to the loss of a single hydroxy group, instead of the two that should have been lost if OssL and OssK were both essential and catalyse different specific hydroxylations. This result also may reflect an overlap of substrate (regio)specificity between the cytochrome P450 hydroxylases. Given this complexity, substantial future work will be needed to clarify their respective roles, and to establish the likely order of the four hydroxylations *in vivo*.

### Iterative use of an extension module in the ossamycin PKS

Complex reduced polyketides are synthesised from simple acyl-CoA precursors on modular polyketide synthases (PKSs) according to an assembly-line paradigm [19,20], in which each cycle of polyketide chain extension is catalysed by a different set or module of fatty acid synthase-related enzymatic activities. Each module of a canonical PKS has minimally a ketosynthase (KS) domain, an acyltransferase (AT) specifying the nature of the incoming extension unit, and an acylcarrier protein (ACP) that tethers intermediates to the assembly line. Depending on the degree of reduction required of the initially-formed β-ketoacyl thioester, each module optionally includes further domains housing ketoreductase (KR), dehydratase (DH), and enoylreductase (ER) activities.

Many modular assembly-line PKS genes are arranged on the chromosome in the order in which their multienzyme products are used for polyketide chain assembly. The eight *oss* PKS genes are not so neatly arranged, but the true order of multienzymes OssA1-A8 in the assembly-line was established to be as shown in Fig 2B, by sequence comparisons with known systems. OssA1 was immediately identified because its N-terminal region contains a loading module comprising a methylmalonyl-thioester decarboxylase (KSQ domain) [36], a methylmalonyl-CoA-specific acyltransferase (AT) and an ACP, which together furnish the propionate starter unit as the substrate for extension module 1. Likewise, OssA8 was identified because its C-terminal region contains a thioesterase (TE) domain that catalyses the closure of the macrocyclic ring and releases the full-length chain from the PKS. OssA1-OssA4 were found to contain a total of eight extension modules, with respective domain complements that exactly matched those required to elaborate the C-15 to C-33 portion of the ossamycin polyketide backbone, as explained below. Likewise, OssA7 and OssA8 between them house four extension modules which match those required to specify synthesis of C-1 to C-8 of the polyketide chain. OssA5 and OssA6 contain one module each, and clearly contribute to construction of the middle part of the polyketide chain, but this gives a total of only 14 extension modules, when 15 are required to produce ossamycin.

To help resolve this discrepancy, domain-by-domain analysis was carried out within each module of sequence motifs in the respective AT, KR, DH and ER active sites to obtain a detailed prediction of the chemical nature and configuration of the polyketide product. The predicted substrate specificity of AT domains was determined by examination of active site sequence motifs (S1A Fig). AT domains selecting malonyl-CoA have the sequence GHSQ around the active site serine residue, and around the second histidine in the active site sequence HAFH. Those domains selecting (2*S*)-methylmalonyl-CoA have the fingerprint residues GHS(I/L/V) and YASH at these positions [37–39]. The pattern of extender unit incorporation during assembly-line biosynthesis on the *oss* PKS matched that expected for ossamycin, with three exceptions. The first of these is that AT2 is predicted to select methylmalonyl-CoA, whereas malonyl-CoA is actually inserted at this position during ossamycin biosynthesis to form C-27 and C-28. Other features of AT2 must account for its preference being shifted, and it is intriguing that in other members of the oligomycin/rutamycin family methylmalonyl-CoA is indeed inserted at the equivalent position (Fig 1). A second exception is in module seven, where AT7 selects the unusual extender unit isobutyrylmalonyl-CoA. Here, the YASH specificity motif is replaced by SPGH. Alterations in this sequence motif to smaller amino acids are typical in other AT domains that recruit exotic alkylmalonyl-CoA substrates. Thus, AT4 in the divergolide PKS, which also selects isobutyrylmalonyl-CoA, has the fingerprint residues VASH at this position [22]. The final exception is that, because only 14 extension modules are present and 15 modules are required, a module must be used twice. This question is addressed more fully below.

The predicted outcome of catalysis by DH, KR and ER domains was likewise determined by examination of active site sequence motifs (S1B, S1C and S1D Fig). In DH domains the essential His residue is embedded in a highly conserved Hxxx(G/D)xxxxPG sequence [40], and an essential Asp in the sequence Dxxx(Q/H) [41], together with other supporting active residues [42]. All the DH domains are predicted to be active except for DH1 and DH7, in which the essential His is substituted by Arg and Tyr respectively. In KR domains, the active site residues important for catalysis include specific Lys, Ser and Tyr residues, with the Tyr residue most important [43]. Other residues in and around the active site correlate with the stereochemical outcome of reduction of the β-carbonyl in the β-ketoacyl-ACP intermediates [43–47]. In A-type KR domains, the resulting hydroxyacyl thioester has the l-configuration, while in B-type KR domains it has d-configuration [44]. A-type KRs have a Trp residue present, while B-type KRs have a specific Asp residue, the third residue in a sequence motif LDD (or similar) [43,44]. When the incoming extension unit is other than malonyl-CoA the configuration of the resulting C-2 asymmetric centre may also be predicted from active site 'fingerprint' residues [47]. All of the extension modules except modules four and seven contain KR domains, and all KR domains are predicted to be active, and to be of either A1, A2 or B1 types, using the nomenclature of Keatinge-Clay [47]. A1-type KRs are predicted to produce a (2*S*,3*R*)3-hydroxy-2-methylacyl-ACP, B1-type to produce a (2*R*,3*R*)3-hydroxy-2-methylacyl-ACP, and A2-type (containing a specific His substitution in place of Gln) to produce a (2*R*,3*R*)3-hydroxy-2-methylacyl-ACP (S1C Fig). In module 11, where full reduction generates an α-methylacylthioester, the product is predicted, from the presence of a specific Tyr-containing motif at the active site [48] (S1C Fig) to be the (2*S*)-stereoisomer. The hypothetical full-length polyketide produced by the *oss* PKS is therefore as shown in Fig 2B.

The structure in Fig 2B fits exactly to the structure of the ossamycin polyketide backbone if chain extensions 9 and 10 are assumed to be carried out by the same monomodular protein (labelled module 9/10 in Fig 2B). This iterative module is proposed to accept the growing chain onto its KS domain as an enoylthioester from module 8, condense it with a malonyl extension unit, and carry out full reduction of the resulting β-ketothioester. The extended chain is transferred back from the ACP to the KS, and the identical elongation cycle is repeated. Finally, the chain is transferred to the KS domain of module 11 for further processing. A number of examples of such programmed modular iteration have been previously described [49–52], although to our knowledge this is the first example of two malonate units being added by a single module.

Significantly, the point in the formation of the macrocycle where iteration is predicted to occur is precisely where the structural difference lies between 22- and 24-membered macrocyclic spiroacetals (Fig 1). It therefore seems highly probable that the PKS biosynthetic machinery for all 22-membered macrocycles will be found to be identical in module number and domain content, the only difference being that iteration does not occur in those assembly lines. During the natural evolution of modular PKSs, programmed iteration offers an additional pathway for increasing the chemical diversity of products from a single assembly-line [53, 54].

### A possible role for OssO in formation of the spiroacetal of ossamycin

The timing of spiroacetal formation remains unknown, and it may occur while the growing polyketide is still attached to the PKS. A separate question is whether the stereospecific spiroacetal formation is spontaneous, or is under enzymatic control. Spiroacetals are a feature of numerous complex polyketides, and several different enzymatic routes to this moiety have been described. In the biosynthesis of reveromycin in *Streptomyces reveromyceticus* the key step involves post-PKS enzymatic oxidation of a hydroxyl group to a ketone by RevG followed by stereocontrolled spiroacetal formation catalysed by RevJ [55]. A similar post-PKS process may account for formation of spirangiens in *Sorangium cellulosum* [56]. However, the structural prediction for the product of the ossamycin PKS implies that the keto group is directly formed at C-25 during elongation on the assembly line. An alternative precedent is offered by the biosynthesis of avermectins in *Streptomyces avermitilis*, where the enzyme AveC (with no structural similarity to RevJ) has been shown to be essential both for stereospecific spiroketal formation from a dihydroxy-ketone polyketide intermediate and for optional dehydration at C22-C23 [57]. Another bifunctional spirocyclase, from biosynthesis of the polyether salinomycin, is the methyltransferase-like SlnM [58] (also known as SalE [59]) whose disruption prevents both formation of a 6,6-spiroacetal and dehydration to give a *cis* double bond at C-18-C-19 [60,61]. Furthermore, formation of the spiroacetal *in vitro* has been directly shown to require SlnM [61].

The *oss* gene cluster does not encode any protein with significant sequence similarity to either RevJ, SalE/SlnM or AveC, and neither is such a protein encoded anywhere else on the *S*. *hygroscopicus* var. *ossamyceticus* chromosome. On the other hand, the cluster has been found to contain a gene (*ossO*), located immediately adjacent to the PKS gene *ossA1*, whose 176 amino acid protein product showed 41% identity with a previously overlooked protein of unknown function encoded in the oligomycin biosynthetic gene cluster of *S*. *avermitilis* (OlmO) [62]. This unexpected finding prompted us to search, using BLAST analysis [63], for other potential homologues of OssO. As a result, seven similar proteins were found, all of which are associated with *Streptomyces* PKS clusters (S2 Fig). Phylogenetic analysis (S3 Fig) showed that OssO-like proteins associated with the authentic oligomycin PKS cluster and with other oligomycin-like PKS clusters form a separate clade from OssO, which groups together with four other proteins from potential macrocyclic spiroacetal PKS clusters. Interestingly, the homologue in the *S*. *pactum* PKS is co-located with the gene cluster for the F_0_F_1_-ATPase, the known target of many macrocyclic spiroacetals. Two other homologues are from the PKS clusters for biosynthetic gene clusters for the polyethers calcimycin [64] and nigericin [65] which both contain spiroacetal moieties. More remote homologues were also detected, for example Aln8 from *Streptomyces* sp. CM020 (ACI88878.1), and SARE_3150 from *Salinispora arenicola* (ABV98958), in PKS-containing clusters for compounds that do not require formation of a spiroacetal. The catalytic role of OssO and OlmO as putative spirocyclases is the subject of ongoing investigation in this laboratory.

## Conclusions

Apart from the oligomycin biosynthetic gene cluster [62] the genes and enzymes for biosynthesis of 22- to 26-membered macrocyclic polyketides bearing a spiroacetal moiety have remained unexplored. We have now identified the biosynthetic gene cluster for ossamycin biosynthesis in the whole-genome sequence of *S*. *hygroscopicus* var. *ossamyceticus* Bioinformatic analysis of the cluster strongly suggests that programmed iteration of a specific extension module on the modular PKSs is required to furnish the 24-membered macrocycle of ossamycin. Such exceptions to the co-linearity rule are rare and offer insight into a possible mechanism for evolution of modular polyketide megasynthases. It is tempting to suggest that the ossamycin PKS evolved from an assembly-line catalysing production of a 22-membered macrocycle (Fig 1) by local mutation within the PKS, allowing a freshly-elongated intermediate to reacylate the KS domain and undergo a second identical cycle of chain extension. It will be of considerable interest to sequence and compare the PKS genes for a naturally-occurring 22-membered macrocyclic polyketide.

The post-PKS hydroxylation pattern in ossamycin biosynthesis shows limited cross-talk between the cytochrome P450 enzymes that hydroxylate the macrocycle. The data do not reveal the timing of the post-PKS processing, but do rule out the targeted enzymes as responsible for the introduction of the hydroxy group at C-8 required for sugar attachment at this position. A potentially novel spirocyclase is also encoded within the ossamycin cluster. The newly identified ossamycin pathway can be a used as a platform for engineering of the new chemicals as useful ATP-inhibitors.

## Methods and materials

### Bacterial strains and culture conditions

*Streptomyces hygroscopicus* var. *ossamyceticus* DSM 40824 (NRRL B-3822) and its mutants were maintained on SFM agar plates (2% soy flour (Arkasoy), 2% d-mannitol, 2% agar, 10 mM MgCl_2_) at 30°C. For ossamycin production DSM 40824 was grown in a TSBY seed medium (3% TSB (Tryptic Soy Broth), 10.3% sucrose, 0.5% yeast extract) at 30°C and 200 rpm on a rotary incubator. After 2 days seed culture (5% v/v) was inoculated into fermentation medium DNPM [66] (4% dextrin, 0.75% soytone, 0.5% wet baking yeasts, and 2.1% MOPS, pH 6.8) and harvested after 7–8 days. *Escherichia coli* strains were grown in Luria-Bertani (LB) broth (1% tryptone, 0.5% yeast extract, 1% NaCl) or agar (1% tryptone, 0.5% yeast extract, 1% NaCl, 2% agar) at 37°C with appropriate antibiotic selection (50 mg/L apramycin, 30 mg/L kanamycin)

### Materials, DNA isolation and manipulation

Bacterial strains, plasmids and oligonucleotides (Sigma) used in this study are summarised in Tables 2, 3 and 4. Isolation of plasmid DNA from *E*. *coli* was performed according to the suppliers' protocols (Promega, Omega Biotek). Phusion^TM^ High-Fidelity DNA (ThermoScientific) and MyTaq^TM^ (Bioline) polymerases were used for DNA amplification. Isolation of chromosomal DNA from *S*. *hygroscopicus* var. *ossamyceticus* DSM 40824 was performed as described earlier [67].

**Table 2:** List of strains used in this study.

Strain	Genotype/characteristics	Reference
*E*. *coli* DH10B	F^-^ mcrA Δ(*mrr-hsd*RMS‐*mcr*BC), Φ80*lacZ*ΔM15, Δ*lac*X74 *rec*A1 *end*A1 *ara*D139 Δ (*ara*, *leu*)7697 *gal*U *gal*K *rps*L *nup*G λ– host for general cloning	Invitrogen
ET12567/pUZ8002	(F^-^ *dam*-13::Tn9 *dcm*-6 *hsd*M *hsd*R recF143 zjj202::Tn10 *gal*K2 *gal*T22 *ara*14 *pac*Y1 *xyl*-5 *leu*B6 *thi*-1) Donor strain for conjugation between *E*. *coli* and *Streptomyces*	[68]
*S*. *hygroscopicus* var. *ossamyceticus* DSM 40824	Wild type strain, producer of ossamycin	[69]
DSM 40824 Δ*ossF*	*ossF* in-frame deletion mutant of the wild type	This work
DSM 40824 Δ*ossF*::*ossF*	Δ*ossF* mutant complemented with the native *ossF*	This work
DSM 40824 Δ*ossLK*	*ossLK* in-frame deletion mutant of the wild type	This work

**Table 3:** List of strains used in this study.

Plasmid	Genotype/characteristics	Reference
pYH7	*E*. *coli*-*Streptomyces* shuttle vector	[70]
pYH7Δ*ossF*	*ossF* deletion plasmid	This work
pYH7Δ*ossLK*	*ossLK* deletion plasmid	This work
pIB139	*E*. *coli*-*Streptomyces* shuttle vector, *attP* (ΦC31), int, P_ermE*_	[71]
pIBossF	*ossF* complementation plasmid	This work

**Table 4:** List of primers used in this work.

Primer	5' to 3' nucleotide sequence	Purpose
dOssF-fF-pYH-f	Ccgggactgatcaaggcgaatacttcagagctccgggtccgtccgcag	deletion of *ossF*
dOssF-fF-r	Tcgccgatgacagggagtcaggaagtggtgaacgcaccgcacacc	deletion of *ossF*
dOssF-fR-f	Gtgggtgtgcggtgcgttcaccacttcctgactccctgtcatcgg	deletion of *ossF*
dOssF-fR-pYH-r	cgggacccgcgcggtcgatccccgcaggcggcgacgaagctgaggga	deletion of *ossF*
dOssLK-fF-pYH-f	Ccgggactgatcaaggcgaatacttcacacgtcctcgtcaacgcggcc	deletion of *ossLK*
dOssLK-fF-r	Gttccttcccgaaggagcgagacgtgcatgctttccttctgtgag	deletion of *ossLK*
dOssLK-fF-r2	Accgtccggttccttcccgaaggatgcatgctttccttctgtgag	deletion of *ossLK*
dOssLK-fR-f2	Cccctcacagaaggaaagcatgcatccttcgggaaggaaccggac	deletion of *ossLK*
OssF-pIB-f	tgccggttggtaggatccaca atggacgtcgccgagaatatc	complementation of Δ*ossF*
OssF-pIB-r	tcctctagaggatccccaaca tcaccacgtgaccggcaggca	complementation of Δ*ossF*
pYH7-NdeI-f	Gctcagggcgacacgatc	pYH7 screening
pYH7-NdeI-r	Ctgaccggcaatcaccaa	pYH7 screening
M13-f	Tgtaaaacgacggccagt	pIB139 screening
M13-r	Caggaaacagctatgac	pIB139 screening

### Metabolites analysis

For analysis of ossamycin production 0.7 ml of the 7 days old *Streptomyces hygroscopicus* var. *ossamyceticus* culture grown in DNPM was extracted with an equal volume of ethyl acetate at 45°C for 1 hour. The mixture was centrifuged for 5 min at 21952 x *g*, the upper phase was evaporated to dryness and dissolved in 100 μl of methanol. 50 μl of extract was analysed by LC-MS. MS analyses were performed on an HPLC (Agilent Technologies 1200 series) coupled to a Thermo Fisher LTQ mass spectrometer fitted with an electrospray ionization (ESI) source. The methanol extracts were loaded onto a Prodigy 5μ C18 column (4.6 x 250 mm, Phenomenex), and the samples were eluted using MQ (+0.1% formic acid, A) and acetonitrile (+0.1% formic acid, B) at a flow rate of 0.7 ml/min. The elution gradient for extracts was 10% to 100% of B over 32 min. The mass spectrometer was operated in positive ionization mode, scanning from m/z 200 to 2000 in full scan mode.

### Inactivation of cytochrome P450 enzymes

All gene knock-outs in *Streptomyces hygroscopicus* var. *ossamyceticus* were performed by introducing an in-frame deletion. 2-kb DNA fragments flanking gene(s) of an interest were amplified using primers carrying 28-bp overlapping regions with pYH7 and each other. pYH7 treated with *Nde*I was ligated with the inserts by isothermal assembly as described previously [72]. The DNA mixture was used to transform *E*. *coli* DH10B. Plasmid DNA isolated from the apramycin resistant colonies was verified by PCR and DNA sequencing to confirm correct insertion of the DNA fragments. The resulting inactivation constructs were introduced into *Streptomyces hygroscopicus* var. *ossamyceticus* by intrageneric conjugation as previously described [73].

### Complementation of *Streptomyces hygroscopicus* var. *ossamyceticus* deletion mutant

The *in trans* complementation of the deletion mutant strain *Streptomyces hygroscopicus* var. *ossamyceticus* Δ*ossF* was performed as follows. The intact *ossF* gene was amplified from the chromosome using ossF_pIB-f and ossF_pIB-r primers and ligated into pIB139 plasmid pre-treated with *Nde*I, using isothermal assembly [19]. The obtained plasmid pIBossF was conjugated into the deletion mutant.

## Supporting information

S1 Fig Active site motifs in the ossamycin cluster of *S*. *hygroscopicus var*. *ossamyceticus*. (DOCX)

S2 Fig Alignment of putative spirocyclases from *Streptomyces* polyketide biosynthetic gene clusters. *S*. *chartreusis*: SchU2 from the calcimycin gene cluster (AEH42486.1) of *S*. *chartreusis* NRRL 3882; *S*. *pactum*: protein B1H29_11740 in the oligomycin-like gene cluster of *S*. *pactum* ACT12 (AQS67518.1); NlmOI, protein in oligomycin-like gene cluster of *S*. *nanchangensis* (AAS46349.1); *S*. *sp*. cf124: protein in uncharacterised PKS gene cluster of *S*. *sp*. cf124 (SFN44164.); *S*. *torulosus*, protein in uncharacterised PKS gene cluster of *S*. *torulosus* NRRL B-3889 (WP_107102773.1); OssO, protein in the ossamycin biosynthetic gene cluster of *S*. *hygroscopicus* var. *ossamyceticus* (MH763624); *S*. *bottropensis*, protein STROBO_RS40610 in uncharacterised PKS gene cluster of *S*. *bottropensis* ATCC 25435 (WP_005483815.1)
(DOCX)

S3 Fig The phylogenetic tree with the highest log likelihood (-2474.38) for the putative spirocyclase family [74]. (TIF)
