# Supplementary material for: The biosynthetic pathway to ossamycin, a macrocyclic polyketide bearing a spiroacetal moiety
Source: PLoS One. 2019 Apr 30;14(4):e0215958. doi: 10.1371/journal.pone.0215958 (PMC6490886; doi:10.1371/journal.pone.0215958)
Supplement: S1 Fig — (DOCX) [file pone.0215958.s001.docx]

a. Ossamycin AT domains

* *

ATLOAD AAVVGHSQGEVAAA...VAV-DYASHSSYVEDVR methylmalonate

AT1 DHLLGHSVGALAAA...LRV-SHAFHSPLMDPML malonate

AT2 DAVMGHSQGEIAAA...VNI-DYASHSPRMAALE methylmalonate

AT3 DVLMGHSLGELVAA...LSV-SHAFHSALMEPML malonate

AT4 DHLLGHSIGELAAA...LKV-SHAFHSPLMEPML malonate

AT5 AAVVGHSQGEIAAA...VEV-DYASHSAHVEHIH methylmalonate

AT6 DVVLGHSLGELVAA...LAV-SHAFHSALMEPML malonate

AT7 DAVVGHSQGEIAAA...IPGAG**SPGH**SPHVEALR isobutyrylmalonate

AT8 DYLFGHSVGEITAA...LTV-GHAFHSPLMEPML malonate

AT9/10 RYLAGHSIGELTAA...LKV-SHAFHSPLMEPML malonate

AT11 AAVVGHSQGEIAAA...VEV-DYASHSAHVEHIH methylmalonate

AT12 DVVFGHSLGEVVAA...LAV-SHAFHSALMEPVL malonate

AT13 AAVVGHSQGEIAAA...IEV-DYASHSVQVEQLR methylmalonate

AT14 AAVIGHSQGEIAAA...IAV-DYASHSAHVEQIE methylmalonate

AT15 DALIGHSVGELVAA...LTV-SHAFHSPLMEPML malonate

b. Ossamycin KS domains

*

KSLOAD VGALADDYARLVQRHGPGAVTPHTF...TVDTGQSSSLVA

KS1 VGAMSQEYGPRLHE-APDELRGHLL...TVDTACSSSLVA

KS2 AGLVAQDYGPRLDE-ASDDTGGHVL...TVDTACSSSLVA

KS3 VGAGHFDYA-ALAL-TTEEGKDYAL...TVDTACSSSLVA

KS4 VGMTDQKYGPEGDE-ALREVRGHVL...SVDTACSSSLVA

KS5 AGIAGSDYA-DVLA-ATPETEGHVM...TVDTACSSSLVA

KS6 VGVSEQGHTARLLD-AATDVEGYFA...TVDTACSSSLVA

KS7 AGVSQQDYATLLTA-TEGRIDGHGS...TVDTACSSSLVA

KS8 VGAAGLGYSLLFPP-GSEQLAGYTV...TVDTACSSSLVA

KS9/10 AGLMYHDYGGELST-LPDEAQGLLS...TVDTACSSSLVT

KS11 LGGGTEDFAGLLAM-CRDAEETASL...TVDTACSSSLVT

KS12 VGGATQEYGALLGD-SPENTDGYAL...TVDTACSSSLVG

KS13 VGGSPTGYGNVVGD-TPD-AGGYLL...TVDTACSSSLVS

KS14 VGGLPTGYGALLMD-SEE-DQGYAL...TVDTACSSSLVA

KS15 VGGTPQEYGALLMN-SPALAGGYAL...TVDTACSSSLVA

**VMYH**

**TNGQ**

c. Ossamycin KR domains

1 2 3 4 5 6

KR1 HTAGVLH**D**AVVEAIDPGDLD... AFVLFSSVSGINGAAGQGSYAAGN B1

KR2 HTAGVSGLERLDTTTPADLA... AFVLFSSGAAV**W**GGARQAAYAAAN A1

KR3 HCAGT**VDD**GVLTSLTPDRVR... SFVLFSSASATFGAAGQANYCAAN B1

KR5 HAAGVGVLGPVVDAPFADLV... AVVYFSSITAV**W**GAGD**H**AVYAAGN A2

KR6 HTAGV**VDD**GVVTALTPERVD... HFVLFSSAVGTLGGAGQANYAAAN B1

KR8 HTAGT**VDD**ATLTALTPEQVD... AFVVFSSLAGTMGGAGQANYAAAN B1

KR9/10 HTAGV**LDD**GIAAGLTDEQLH... VFALFSSASGFTGNPGQANYAAAN B1

KR11 HCAGTT**DD**GALGSLTEERVE... QLLLFSSAAATVGSPGQANYAAAN B1

KR12 HAAGVVRYTKVRDLTPEEID... AFVLFSSGAAS**W**GGGSQGAYAAAN A1

KR13 HAAGVATFSEVLSIEPRELA... AFVLFSSGAAV**W**GSAGNGTYAAAN A1

KR14 HTASSTAYGPVLDIEARDFA... AFVLFSSGAAV**W**GSAGNGTYAAAN A1

KR15 HLAGV**VDD**GLVGDLTAERLT... AFVLFSSAAGVLGSPGQANYAAAN B1

d. Ossamycin DH domains

* * ** ** *** * * * *** *

DH1 AD***R***TVHDHVSVPDSV...PAAGVARGPGFAGLR...HPGLLECVSHAVRLARK...--DRAW**H**GAAYRHV

DH3 ADHTVLGTALFPGTA...IDAGFDYGPAFQGLR...HPALLDAALHALGVDL-...PGTPARLPFAWRGV

DH7 AG***Y***RIGDQAVLPGTA...AAAGFAYGPERRLLG...HPALSEAALHALYALGD...-SRDMAVPFSWGGV

DH8 TDHAVLDTPLFPGTG...IDHGFTYGPAFRGLT...HPALLDAALHALGVDL-...PGTPARLPFAWRGV

DH9 ADHAVGGTVLVPGTA...SAGGFAYGPAFQGVR...HPALADATLHAAVFAGG...AAGQARLPFVWDGV

DH11 ADHVVQGTVLLPGTA...ATAGFDYGPAFRGLR...HPALLDGALHGASLLPG...-DGGARLPFTWSGV

DH15 AEHTLNGVPVVPGTA...AAAGLDYGPVFQGLA...HPALLDAALQPLALGIL...TPVPPGLPFAWSGL

e. Ossamycin ER domains

ER3 FRDTMIALDM**Y**P

ER9/10 FRDVVVALGL**V**P

ER11 FRDTLIALGM**Y**P (2S)

Erymod FRDVLIALGM**Y**P (2S)

Rapmod2 FRDV-WALGM**V**N (2R)
