# Supplementary material for: The biosynthetic pathway to ossamycin, a macrocyclic polyketide bearing a spiroacetal moiety
Source: PLoS One. 2019 Apr 30;14(4):e0215958. doi: 10.1371/journal.pone.0215958 (PMC6490886; doi:10.1371/journal.pone.0215958)
Supplement: S2 Fig — S. chartreusis: SchU2 from the calcimycin gene cluster (AEH42486.1) of S. chartreusis NRRL 3882; S. pactum: protein B1H29_11740 in the oligomycin-like gene cluster of S. pactum ACT12 (AQS67518.1); NlmOI, protein in oligomycin-like gene cluster of S. nanchangensis (AAS46349.1); S. sp. cf124: protein in uncharacterised PKS gene cluster of S. sp. cf124 (SFN44164.); S. torulosus, protein in uncharacterised PKS gene cluster of S. torulosus NRRL B-3889 (WP_107102773.1); OssO, protein in the ossamycin biosynthetic gene cluster of S. hygroscopicus var. ossamyceticus (MH763624); S. bottropensis, protein STROBO_RS40610 in uncharacterised PKS gene cluster of S. bottropensis ATCC 25435 (WP_005483815.1) (DOCX) [file pone.0215958.s002.docx]

*S. chartreusis* MT--DIASDAGKINTYLSGRPTERPRTEHRPPSPRLKDLDFLLGRMVTHFDTGVRM---- 54

OlmO MSV----------SEKEAETSAEQEF-TRPPAPEKMRDLDFLLGDFRAEWTNFTADPATT 49

*S. pactum* MSI----------SEQESTPSGEQQF-TKPPAPEKMTDLNFLLGDFRAEWTNFTADPPTK 49

NlmOI MGV----------FEQEAAESTGEKF-VRPAAPERMRDLDFLLGDFRVEWTNFTADPPVK 49

*S. sp.* cf124 MSAQPVQPSDEVMRDSLPADSGEVVFPGRPDVAPEMKQLDFLLGDFRVEYTNFTTEKVTT 60

*S. torulosus* MSAQPIQSSDDASPDSDPIGSGAVVLPSRPDIAPEMRQLDFLLGDFRIEYTNLTTEDITT 60

OssO MSAQPIQSSDDALRDSVPAESGEVAFPGRPDVAVEMRQLDFLLGDFRIEYTNLTTETVTT 60

*S. bottropensis* MSAQPIQSSDDALRDSVPAASGEVVMPGRGEPAPEMRQLDFLLGDFRIEYTNLTTEMVTT 60

* : .: :*:**** : .: . .

*S. chartreusis* ---ESVTRRVMHDHYIQMDLTGTYADGTWKLDGKWIIGWNEVQQQFESYYIDTMGTMGTV 111

OlmO GTAAWNTASTFHGHAYEMTQR----VEAHDLTGRFVVQWVESESSFSGYYYDDWGNRTLL 105

*S. pactum* GIASWNTASTFSGHAYEMTQR----VPEHDLTGRFVVQWVESDSAFSGYYYDDWGNRTLL 105

NlmOI GTAAWNTVSTFAGHAYEMTQL----VPKDDLTGRFVIQWVESESSFSGYYYDDWGNRTLL 105

*S. sp.* cf124 GEATWSTHPVAEGRFLHMTQT----IPVPGIVADWLFGWSEVDSAFICFYYDDWGNHGTF 116

*S. torulosus* GQATCSARSLADGRFLELTQR----IPVPGIVATWLLGWSDVDKSFVSLYYDDWGHHGTF 116

OssO GEATCSTRPLADGRFYELTQR----VPVPGLVATWLIGWSDVDNRFVSFYYDDWGHHGRF 116

*S. bottropensis* GEATCSTRSLADGRFHELTQR----IPVPGIVATWLIGWSDVDKAFTSFYYDDWGHHGVF 116

: .: .: : . ::. * : :. * * * * .

*S. chartreusis* TSPGWQEDGHLVFSGFCVLGEVGVRTNTKDVYTVLGDHHFQLDAYVEAE--GEWKHYDTQ 169

OlmO TSEGWQ-DGYLAFTGECFG--FGKSFLLKEQYEIVDEKHYVKRGFIKFD-EGDWIPADEV 161

*S. pactum* TAGGWE-DGRLAFVGECIG--FGKTFLLKERYEIVDENHYVKRGFVKFEENGDWIPADEV 162

NlmOI TAKGWQ-DGYLSFTGECIG--FGRWFLLKERYQVIDENHYLKCGFIRFEADGEWVPADEV 162

*S. sp.* cf124 TSPGWA-DGRFRVSGDSAV--FGGRHSFVDDFSVVDADHFFKKGFIAVG--DDLVQGDEM 171

*S. torulosus* TGPGWV-DGHFRLKGDSAV--FGSRHVFVEDFEIVDSDHLVKHGFIVVG--DDLVPGDIL 171

OssO TGPGWV-DGHFKLTGDSAV--FGARHGFVEDFEIVDSDHLVKHGFVVVG--DDLVPGDIL 171

*S. bottropensis* GGPGWV-DGHFRLTGESAV--FGARHGFVEDFEVIDDDHIVKHGFVAVG--DELVPGDIL 171

. ** ** : . * . .* : : ::. .* .:: .: *

*S. chartreusis* DCHVTPEQQGS 180

OlmO HCHREA----- 167

*S. pactum* HCYRD------ 167

NlmOI HCYRV------ 167

*S. sp.* cf124 HCYRI------ 176

*S. torulosus* HFHRI------ 176

OssO HFHRI------ 176

*S. bottropensis* HFHRI------ 176
