# Supplementary figures and images for: The biosynthetic pathway to ossamycin, a macrocyclic polyketide bearing a spiroacetal moiety
Source: PLoS One. 2019 Apr 30;14(4):e0215958. doi: 10.1371/journal.pone.0215958 (PMC6490886; doi:10.1371/journal.pone.0215958)

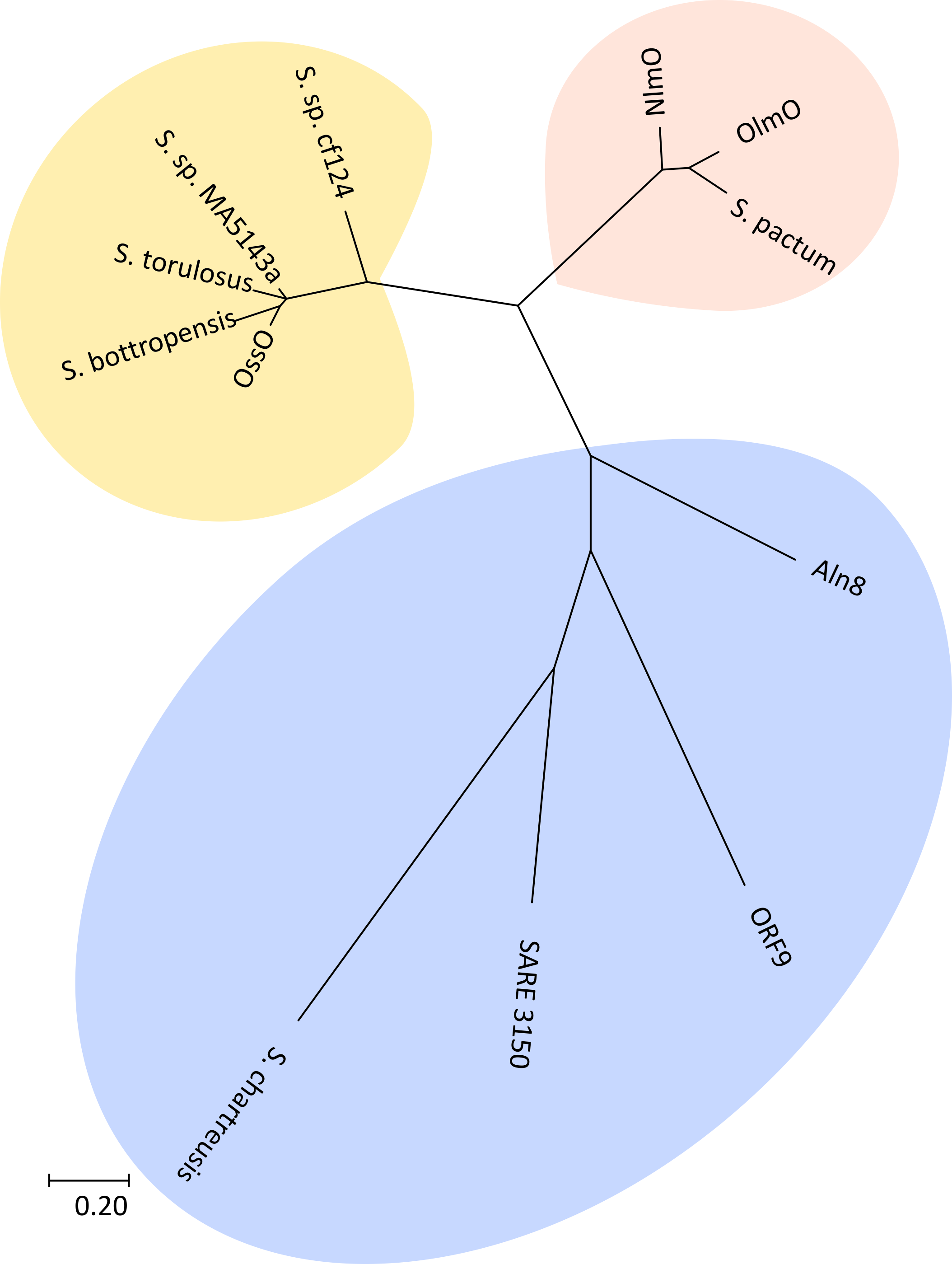

Supplement: S3 Fig — (TIF) [file pone.0215958.s003.tif]
